# Current comprehensive understanding of denosumab (the RANKL neutralizing antibody) in the treatment of bone metastasis of malignant tumors, including pharmacological mechanism and clinical trials

**DOI:** 10.3389/fonc.2023.1133828

**Published:** 2023-02-13

**Authors:** Junjie Lu, Desheng Hu, Yan Zhang, Chen Ma, Lin Shen, Bo Shuai

**Affiliations:** ^1^ Department of Integrated Traditional Chinese and Western Medicine, Union Hospital, Tongji Medical College, Huazhong University of Science and Technology, Wuhan, China; ^2^ Department of Pain, Union Hospital, Tongji Medical College, Huazhong University of Science and Technology, Wuhan, China

**Keywords:** denosumab, RANK/RANKL/OPG system, bone metastasis, skeletal-related events, osteoclast

## Abstract

Denosumab, a fully humanized monoclonal neutralizing antibody, inhibits activation of the RANK/RANKL/OPG signaling pathway through competitive binding with RANKL, thereby inhibiting osteoclast-mediated bone resorption. Denosumab inhibits bone loss; therefore, it is used to treat metabolic bone diseases (including postmenopausal osteoporosis, male osteoporosis, and glucocorticoid-induced osteoporosis), in clinical practice. Since then, multiple effects of denosumab have been discovered. A growing body of evidence suggests that denosumab has a variety of pharmacological activities and broad potential in clinical diseases such as osteoarthritis, bone tumors, and other autoimmune diseases. Currently, Denosumab is emerging as a treatment for patients with malignancy bone metastases, and it also shows direct or indirect anti-tumor effects in preclinical models and clinical applications. However, as an innovative drug, its clinical use for bone metastasis of malignant tumors is still insufficient, and its mechanism of action needs to be further investigated. This review systematically summarizes the pharmacological mechanism of action of denosumab and the current understanding and clinical practice of the use of denosumab for bone metastasis of malignant tumors to help clinicians and researchers deepen their understanding of denosumab.

## Introduction

### The RANK/RANKL/OPG system The Receptor activator of NF-kB/The Receptor activator of NF-kB ligand/Osteoprotegerin system

The receptor activator of NF-kB ligand (RANKL was originally defined as a new member of the tumor necrosis factor receptor (TNFR) family which is expressed on non-dendritic cells and participates in dendritic cell-mediated T cell proliferation and RANK+T cell activation (1). The discovery of RANKL built a bridge between the bone and the immune system and became an important landmark in the rise of bone immunology (2–4). As the RANKL/RANK (Receptor activator of NF-kB**)** signaling pathway plays an important role in mediating osteoclast differentiation and function (5), the relationship between RANKL and bone metabolism has been extensively studied.

The differentiation and maturation of osteoblasts is regulated by two systems: the RANK/RANKL system and the macrophage colony-stimulating factor/colony-stimulating factor-1 receptor (M-CSF/c-FMS) system (6). The M-CSF/c-FMS system is responsible for regulating the differentiation of early hematopoietic stem cells (HSCs) into osteoclast precursor cells and the survival of osteoclast precursor cells (7), whereas the RANK/RANKL system is an important trigger for the differentiation of osteoclast precursors into functional osteoclasts. RANKL is a homologous trimeric transmembrane protein which has two receptors: the membrane-binding receptor RANK and the soluble bait receptor OPG (8). In bone, RANKL is expressed in the bone matrix, osteoblast precursor cells, and osteoblasts, and RANK is expressed on the membrane surface of osteoclasts and osteoclast precursors as a membrane-binding receptor (9).

**Figure 1:**
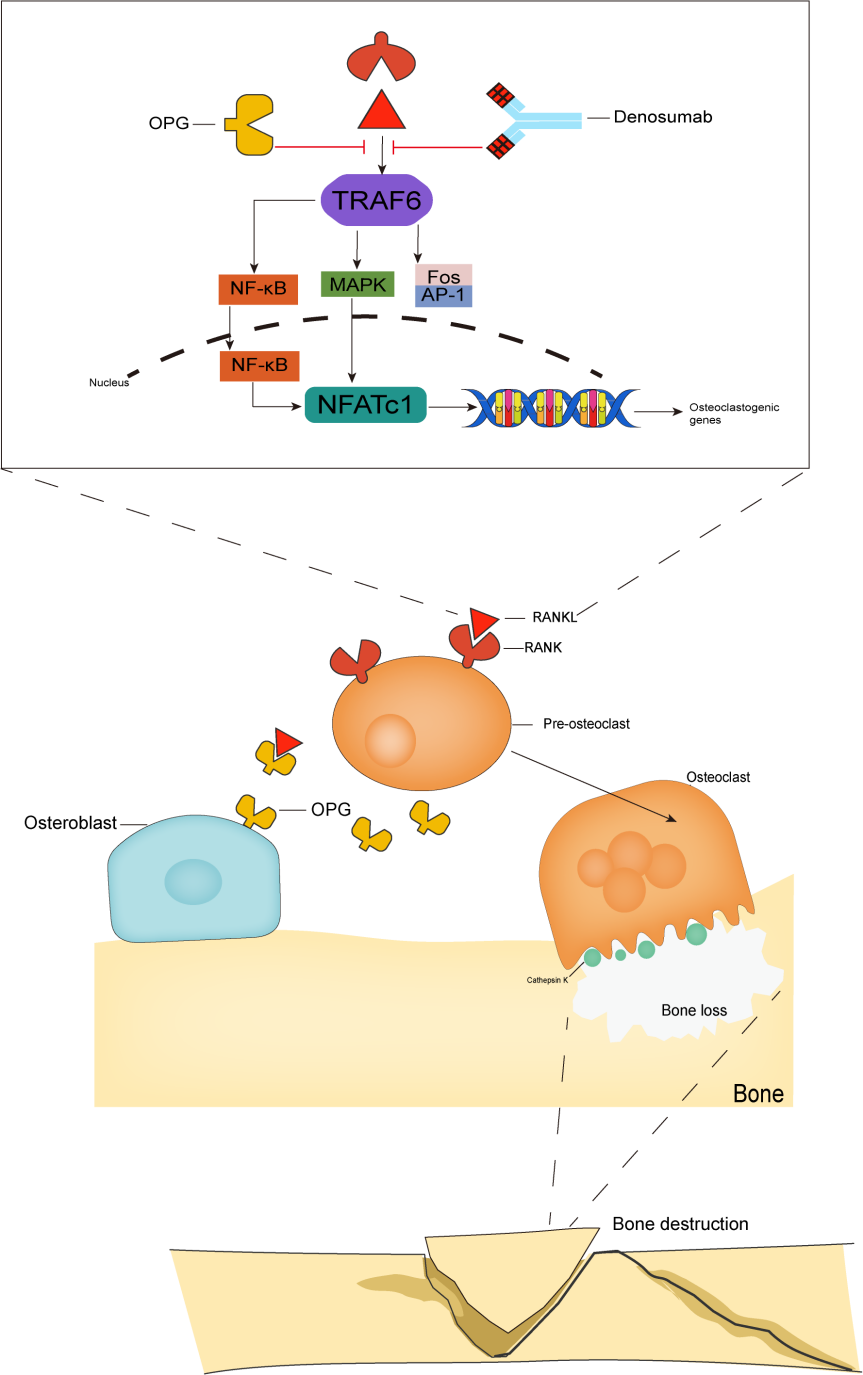
The RANK/RANKL/OPG system. The binding of RANKL to RANK leads to TRAF6 recruitment, which activates NF-kB, MAPK and Fos/AP-1 pathways. These activating signals together lead to the activation of NFATc1, a key transcription factor for downstream osteoclast-associated gene activation, which is the hallmark event of osteoclast formation. The OPG competitively binds RANKL and thus inhibits osteoclast activation, while denosumab, which has the same molecular weight as OPG, also binds to RANKL and inhibits osteoclast activation and maturation.

The binding of RANKL to RANK leads to the recruitment of TNF receptor associated factor 6 (TRAF6) as an articulatory molecule, which activates the NF-kB, c-Fos/AP1, MAPK, and other signaling pathways (10), leading to increased activation, amplification, and transcription of the downstream signal nuclear factor of activated T cells (NFATc1) (11), which directly mediates the differentiation of osteoclast precursor cells into osteoclasts (12). NFATc1 is a major regulator of osteogenesis (13). NFATc1 is both a major regulator of osteoclast formation and is involved in the regulation of osteoclast-specific genes (TRAP, Cathepsin K, calcitonin receptor) involved in osteoclast differentiation proliferation and survival (14, 15). The osteeoprotegerin (OPG) inhibits the activation of RANK signaling by competitively binding to RANKL and preventing RANKL from binding to its receptor RANK (16) (**Figure 1**).

Denosumab is the first and only clinically available RANKL inhibitor that inhibits osteoclast activity by targeting and blocking the binding between RANK and RANKL. While inhibiting the function of mature osteoclasts (17), it also inhibits the maturation of osteoclast precursor cells, reduces bone resorption, and promotes bone reconstruction, thereby delaying bone-related events.

### Biology of bone metastasis of malignant tumors

**Figure 2:**
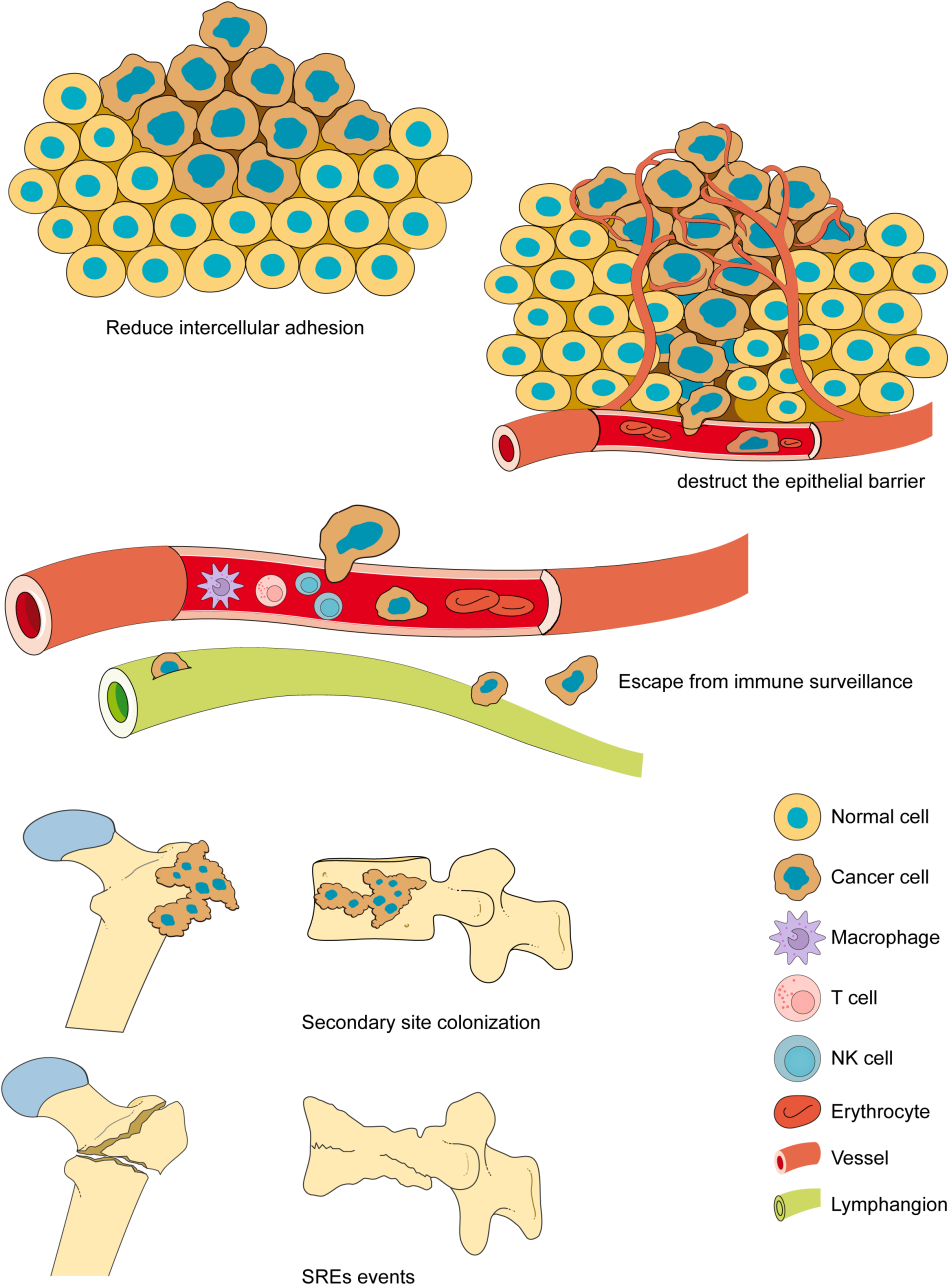
The bone metastasis. The bone metastasis is a complex biological process. Tumor cells metastasizing from the primary site to other tissues and organs generally undergo the steps of reducing intercellular adhesion, overcoming barriers, escaping from immunity, colonizing secondary sites, proliferation and growth and lead to SREs. The bone metastases often lead to serious complications, including fractures, nerve compression, and severe pain, resulting in loss of mobility, a significant increase in medical costs, and a significant reduction in quality of life and survival.

Tumor metastasis is the process by which cancer cells spread from a primary lesion to other sites. Cancer cells metastasize in three major ways: direct invasion, disseminated metastasis, and vascular and lymphatic metastases (18). Tumor metastasis is a complex biological process (19, 20). Tumor cells metastasizing from the primary site to other tissues and organs generally undergo the steps of reducing intercellular adhesion, destructing the epithelial barriers, escaping from immune surveillance, secondary site colonizing, proliferation and growth and lead to skeletal-related event SREs (21) (**Figure 2**). The three major primary cancers that are most prone to bone metastasis are breast, lung, and prostate cancers (22). Tumor cells colonize the bone microenvironment from the primary site, resulting in bone disease, which is defined as a SREs. Although all are bone metastases, the different origins of the tumors lead to completely opposite characteristics. When osteoblast-mediated bone formation predominates, the bone shows abnormal proliferation and presents with osteosclerotic malignancy; when osteoclast-mediated bone resorption predominates, the bone shows abnormal resorption and presents with osteolytic malignancy (23), and there are also some mixed lesions in which osteosclerosis and osteolysis abnormalities occur simultaneously (24).

Tumor progression or invasion of other tumors leads to the disruption of bone homeostasis, forming a vicious circle between osteoclasts, osteoblasts, immune cells, and tumor cells (25). Malignant tumors release a variety of cytokines that can directly or indirectly activate osteoblasts or osteoclasts. When tumor cells secrete IL-1 (Interleukin-1), IL-6 (Interleukin-6), TNF-α (Tumor Necrosis Factor alpha), and other inflammatory cytokines, they activate osteoclasts in large quantities, leading to enhanced osteolysis activity, which produces inflammatory cytokines in large quantities, forming a vicious circle in the bone microenvironment promoting pro-tumor transformation and tumor cell progression (26). In addition, the alteration of the bone microenvironment and tumor microenvironment will make the immune cells’ surveillance and clearance effect on the tumor weaken (27). Tumor cells will block the immune response in many ways, resulting in the weakening of immune cell anti-tumor immunity (28). Physicochemical and environmental factors also play an important role in regulating the progression of tumor metastasis. A hypoxic environment and low pH values are conducive to tumor cell proliferation (29), and this hypoxic acidic environment creates a suitable environment for tumor cell growth, leading to increased levels of tumor cell production, migration, invasion, and proliferation (30).

Bone metastases often lead to serious complications, including fractures, nerve compression, and severe pain, resulting in loss of mobility, a significant increase in medical costs, and a significantly lower quality of life and survival rates (31). Metastases occur in approximately 50% of patients with tumors and are the cause of death in 90% of patients with cancer (32). In the past decades, metastases have been treated using systemic approaches, including chemotherapy and immunotherapy, but most patients with new or recurrent metastases still die within 5 years of diagnosis (33). This is especially true for the high incidence and high risk of bone metastases, so there is an urgent need to explore in depth the options to prevent and treat bone metastases (34). Bisphosphonates are well documented (35). Recognizing the development of early metastases in women suffering from breast cancer, which usually occur in bone tissue, attempts have been made to use bisphosphonates for early prevention in women with breast cancer as a nonspecific treatment, decreasing the potential impact of SREs and increasing the overall survival benefit (36).

### The RANK/RANKL system in tumor metastases to bone

RANKL has been recognized for its role in mediating dendritic cell survival and T cell proliferation, and subsequently for its crucial roles in mediating osteoclast differentiation and function (37). Thus, it has been intensively studied in the field of bone metabolism, and recently, research has returned to focus on the immune system. Many studies have shown that the RANK/RANKL system plays an essential role in developmental maturation and functional maintenance of the immune system. By genetically engineering RANK- or RANKL-deficient mice, it has been found that RANKL knock out mice show lymph node deficiency and impaired B-cell development (38, 39), and patients with mutations in the TNFRSF11A gene (encoding RANK) show a significant reduction in B-cell numbers (40), which confirms that RANK/RANKL is essential for early T cell and B cell development. In addition, RANK/RANKL intervenes in the interactions between T cells and dendritic cells s, and RANKL enhances dendritic cell formation and function in the absence of co-stimulation and antigen presentation, enhancing the ability of dendritic cells to stimulate the proliferation and differentiation of naive T cells (41). In addition, RANK/RANKL activation triggers intracellular signaling pathways (e.g., MAPK, NF-kB, Fos/AP-1, JNK/ERK/P38), which are involved in tumor proliferation and metabolic activities (42).There are many preclinical studies on the above-mentioned signaling pathways in RANKL activation-induced cancer metastasis. For example, MAPK pathway is involved in RANKL-induced breast cancer cell migration, and inhibition of MAPK pathway activation by specific inhibitors can effectively block RANKL-induced cell migration (43, 44). RANKL induces NF-KB activation leading to enhanced aggressiveness of oral squamous cell carcinoma by suppressing RANKL expression, which inhibits RANKL-induced NF-KB activation thereby suppressing the invasion of oral squamous cell carcinoma into the jawbone (45). However, these intracellular signaling pathways do not exist in isolation, but in crosstalk with each other (46). Until now, evidence on the crosstalk between RANK/RANKL and intracellular signaling pathways to regulate tumor proliferation and metabolism is still incomplete. and needs further study.

Although it is not clear whether the RANK/RANKL signaling pathway plays a favorable or unfavorable role in tumor proliferation and metabolism, there is no doubt that RANK/RANKL signaling plays a very important role in tumors. RANK/RANKL is expressed in many tumor tissues (39), and many breast cancer patients show abnormally high levels of RANKL expression in primary lesions, and a positive correlation with the incidence of bone metastases (47). RANK/RANKL is directly involved in tumor proliferation and metabolism and regulates the tumor immune microenvironment (48). The activation of dendritic cells releases a large amount of activated cytokines (including IL-1, IL-6, and IL-12) (41), which increase the number of transcriptional factor Foxp3 regulatory T cells (Foxp3+ Tregs) (49), and induce the differentiation of CD4+ T cells into Th1 cells (50), all of which lead to immunosuppression, and allow tumor cells to escape immune surveillance that promotes tumor progression.

The above evidence seems to indicate a negative aspect of the RANK/RANKL system in the progression of tumors and anti-tumor immunity. Because of the effectiveness of osteoclast inhibition in preventing bone metastases, drugs acting on the RANK/RANKL system have been developed and used to treat bone metastases from malignant neoplasm.

### The development and pharmacological mechanism of denosumab

OPG was discovered in the 1990s when genomics was developed and used for target identification and Amgen discovered emerging mRNAs through large-scale sequencing and studied the function of these genes *in vivo* by overexpressing them in mouse liver. Mice with OPG transfer gene show a phenotype of increased bone density in the lower limb bones. Following the discovery of the OPG phenotype, subsequent studies began to search for a ligand for OPG. The OPG ligand (OPGL) was screened by fluorescence techniques, and a series of subsequent studies revealed that the OPGL sequence was identical to the RANK ligand RANKL, which was then used as a ligand for OPG. Because of its phenotype of increasing bone density, OPG was used to inhibit bone resorption. Hundreds of variants of OPG were developed and used in preclinical animal models, but all showed poor bioactivity and poor pharmacokinetics, and the subsequent OPG immunoglobulin Fc fusion protein (OPG-Fc) had an extended potency enhancer half-life but presented safety risks in phase I trials. Development of OPG-Fc was discontinued and shifted to RANKL. Amgen reconstituted a fully human monoclonal antibody, denosumab, using a modified Ig2 antibody (the modified Ig2 antibody enhances resistance to papain and thus improves efficacy) with little or no cytotoxicity (antibody-dependent cell-mediated cytotoxicity and complement-dependent cytotoxicity) and improved pharmacokinetics (51).

**Figure 3:**
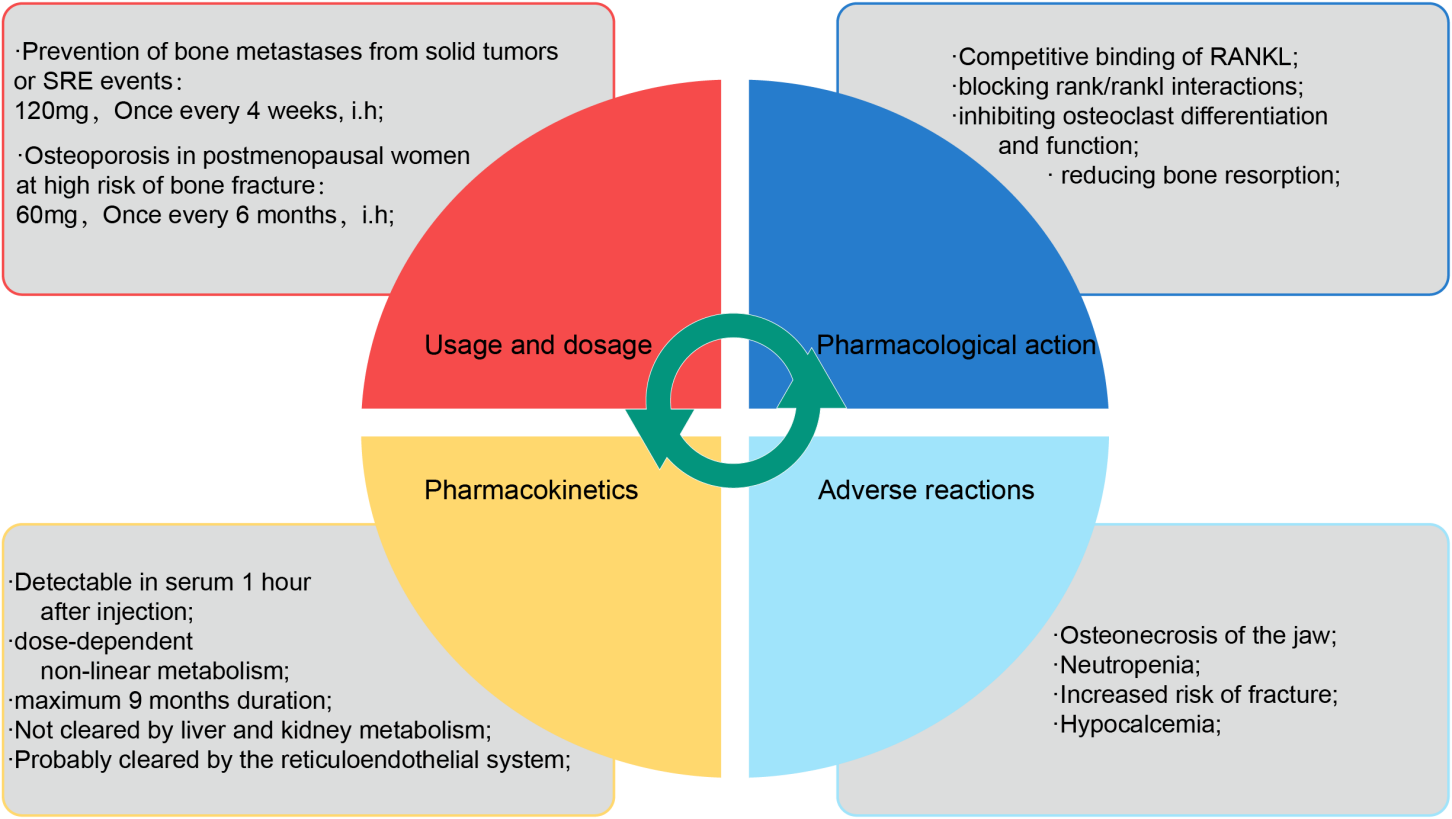
The description of denosumab. The properties of denosumab are briefly described in four dimensions: usage and dosage, pharmacological mechanism, pharmacokinetics and adverse reactions.

Denosumab is a fully synthetic monoclonal neutralizing antibody that acts as an IgG2 subclass immunoglobulin, inhibiting osteoclast differentiation, survival, and activity by competitively binding RANKL, thereby blocking RANK binding to RANKL. Denosumab is considered a highly effective inhibitor of osteoclast bone resorption (52). *In vitro* studies have shown that denosumab, similar to OPG, has high affinity for soluble and membrane-bound RANKL (53). Denosumab has good pharmacokinetic properties, and although there are individual metabolic differences, its molecular mass and structural properties allow for rapid absorption and a nonlinear metabolic profile that can be sustained *in vivo* (54). After subcutaneously administration denosumab of 60 mg, maximum serum denosumab concentration was reached on day 10 (range: 2-28 days), with serum levels declining gradually over 3 months (range: 1.5-4.5 months), with a half-life of 26 days (range: 6-52 days). After subcutaneously administration denosumab of 120 mg every 4 weeks, steady-state concentrations were achieved at 6 months. the mean (± standard deviation) serum steady-state trough concentration at 6 months was 20.5 (± 13.5) µg/mL. The mean elimination half-life was 28 days. It can last up to 9 months after a single dose (55). Similar to other monoclonal antibodies, denosumab is likely cleared *in vivo* by the reticuloendothelial system and is not metabolized by the liver or kidneys (56); therefore, no further impairment of renal function or changes in efficacy or pharmacokinetics have been reported with denosumab in clinical trials, including renal replacement therapy in patients with impaired renal function (57). However, pilot studies on the mechanism of clearance of denosumab, and clinical evaluation of hepatic impairment on the efficacy and pharmacokinetics of denosumab have not yet been conducted (**Figure 3**).

### The clinical trials of denosumab on bone metastasis of malignant tumor

**Figure 4:**
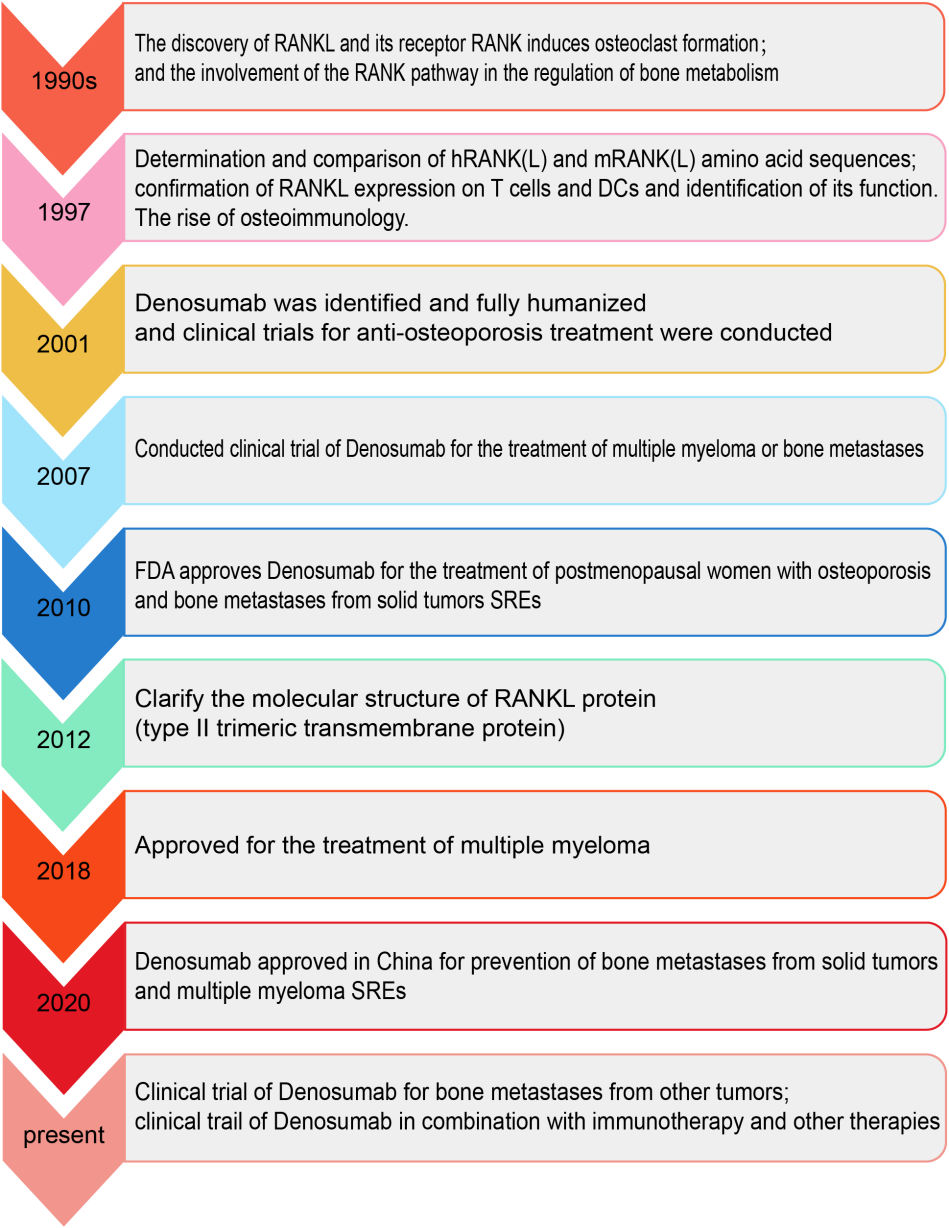
The timeline of RANK/RANKL/OPG system and denosumab. The chronological order shows the important events in the process from the discovery of the RANK/RANKL/OPG system and denosumab to its clinical use.

The RANKL inhibitor denosumab has entered clinical trials for malignant bone metastases (**Figure 4**). A prospective double-blind placebo-controlled phase III trial (58) with 3425 subjects showed that denosumab in women with early-stage hormone receptor-positive postmenopausal breast cancer treated with an aromatase inhibitor was effective in reducing bone mineral density and fractures due to aggressive bone resorption, with no significant variation in the incidence of adverse events (58). Another prospective double-blind placebo-controlled phase I trial with 1432 subjects showed that denosumab was effective in preventing bone metastases in non-metastatic castration-resistant prostate cancer, and that denosumab significantly postponed the onset of first bone metastasis in patients with this type of tumor (59). A randomized phase II clinical study showed that in patients experiencing bone metastases associated with malignant tumors, including prostate, breast, or other tumors, who received bisphosphonates by intravenous injection but still had excessive bone resorption (urinary N-terminal peptide uNTx >100 nmol), increased treatment with denosumab was effective in reducing uNTx levels, inhibiting the bone resorption rate, and reducing the incidence of SREs (60). In addition, a double-blind randomized phase III clinical trial showed that treatment with denosumab (120 mg every 4 weeks) prolonged the time to first bone metastasis radiotherapy compared to conventional bisphosphonate anti-bone metastases (61), implying that the time-lapse to bone metastases was delayed in patients receiving denosumab subcutaneous injections, in addition to prolonging the time to the first SREs occurrence and hypercalcemia, reduced pain levels, and improved the well-being of people suffering from bone metastases. In addition, denosumab effectively reduced serum calcium levels in patients with refractory hypercalcemia whose serum calcium could not be controlled with intravenous bisphosphonate therapy (62), achieving an overall remission rate of 64%, delaying the onset of hypercalcemia in patients with advanced bone metastases, achieving a durable therapeutic response in reducing serum calcium, and being used in patients with renal failure (compared to bisphosphonates). Denosumab is neither metabolized nor excreted by the kidneys compared to bisphosphonates), making denosumab a promising second drug to be approved for the treatment of refractory hypercalcemia after zoledronic acid.

The use of denosumab has produced alterations in bone metabolism and therefore its application in some specific skeletal metabolic disorders deserves to be noted. Paget’s disease is characterized by local bone metabolism disorder, partial bone overgrowth, disorder of bone reconstruction, abnormal osteoclast metabolism causing bone lysis, compensatory increase of osteoblasts, brittle change of abnormally proliferated bone tissue, bone expansion and loosening, and easy fracture, so some clinical studies abroad use denosumab to intervene in early Paget’s disease. In two reported clinical cases (63, 64), patients with Paget’s disease treated with bisphosphonates for a long time, who progressed to giant cell tumor of bone, received subcutaneous injections of denosumab (120 mg every 4 weeks), and imaging showed a reduction in tumor size and an improvement in clinical symptoms. Treatment of bone metastases is usually systemic, and radiotherapy and chemotherapy are the conventional means of treatment for bone metastases. Patients with bone metastases have usually undergone systemic treatment prior to the development of bone metastases, does the combination of denosumab and chemotherapy produce a synergistic effect? Does the combination of denosumab and chemotherapy have a synergistic effect? Does it have an effect on effectiveness or does it produce drug resistance? Studies in animal models have shown that inhibition of RANKL improves the efficacy of the chemotherapeutic agent cisplatin, but there are no objective data on the effect of denosumab in combination with chemotherapeutic agents at this time (39).

Based on the above clinical study results, denosumab could be considered for use as a more effective anti-bone metastasis drug than bisphosphonates, because it delays the onset of bone metastases, reduces the frequency of SREs, improves patient life treatment, and can also be used in patients with bone metastases who are allergic to bisphosphonates or have renal failure (65). However, caution should be exercised. The clinical application of drugs is different from preclinical studies, because tumorigenesis is a complex process with differences in the nature of the tumor itself, and tumorigenesis leads to systemic metabolic changes. There are already relevant clinical studies that are skeptical of denosumab for bone metastases from malignant tumors.

A large multicenter prospective randomized clinical trial revealed the effect of adjuvant treatment with denosumab on early-stage female breast cancer patients. A total of 4509 female breast cancer patients were enrolled in the study (66), half of whom received denosumab (120 mg once a month) at the start of chemotherapy for five years, primarily to determine whether denosumab could play an anti-metastatic role. Unfortunately, there was no significant difference between the two groups, and no significant improvement or therapeutic effect on bone metastases, in addition to neutropenia in 15% of patients and osteonecrosis of the jaw in 5% of patients. Another randomized open phase III clinical study showed that adding denosumab to standard first-line platinum-based dual therapy did not improve overall survival in patients with advanced non-small cell lung cancer (NSCLC) (67). These clinical trials showed that the combination of denosumab did not benefit patients and imposed a financial burden on these patients. A large randomized trial showed a 9-fold increase in medical costs for monthly denosumab treatment compared with 3-monthly zoledronic acid treatment, but no significant survival extension or other benefits were observed (68).

In addition, the increased incidence of adverse events associated with the use of denosumab cannot be ignored, with frequently reported adverse events being osteonecrosis of the jaw, neutropenia, and increased risk of fracture (61, 69).

Based on the above studies, we must carefully evaluate the use of denosumab for the prophylaxis and treatment of bone metastases in malignant tumors. The following questions require careful consideration;

- Whether denosumab is only indicated for certain specific tumor types?
- Whether the use of denosumab brings more clinical benefits and medical cost savings to patients with malignant tumors?
- Whether the combination of denosumab with first-line chemotherapeutic agents may superimpose adverse effects and how to prevent rebound effects after denosumab discontinuation are worthy of prudent evaluation.

Therefore, joint efforts by researchers and clinicians are required.

## Conclusion

In 2020, there will be approximately 19.3 million new cancer cases and 10 million cancer deaths worldwide, and with the growing population base and aging population, this number is expected to increase by 47% by 2040, with the global cancer burden reaching 28.4 million cases (70, 71). Approximately 50% of patients with tumors develop metastases, and the majority of patients with tumors die from a variety of complications caused by metastases, rather than from other causes. Bone metastatic disease is most common in some specific cancers, among which the incidence of bone metastasis in breast cancer is approximately 70%, prostate cancer is about 85%, cancer bone metastasis is about 40%, and the incidence of bone metastasis in multiple myeloma is as high as 95% (22). Given the high incidence of these tumors, many bone metastases occur each year, causing great pain and devastation to patients. Most bone metastases occur in the spine, pelvis, ribs, and other important areas (72), causing pain, compression, bone destruction, pathological fractures, and other serious SREs (73). Approximately 40% of patients with bone metastases experience SREs during antitumor treatment (73).

The evolution of malignant tumor metastasis to the bone is a complex process (74). The metastatic spread of tumor cells includes reducing intercellular adhesion, destructing the epithelial barrier, escaping from immune surveillance, secondary site colonization and SREs events happening (75). However, when tumor cells colonize the bone, it also provides favorable support for the rapid proliferation of tumor cells. The relationship between tumor cells and the bone microenvironment has been compared to the relationship between seeds and soil (76), and the various cells and blood supply in the skeletal system provide a natural breeding ground for tumor cells to colonize and proliferate. However, not all types of tumors develop bone metastasis, and it seems that the characteristics of the seed (i.e., tumor cells) interacting with the soil (i.e., bone microenvironment), play a more important role in the spread of malignant tumor bone metastasis (77), which may explain why some specific primary tumor types (e.g., breast cancer and prostate cancer), are more prone to bone metastasis (78). In addition, differences in the primary foci classify bone metastases into different types, and bone metastases are classified into osteosclerotic malignancies, osteolytic malignancies, and mixed malignancies (79). Osteolytic malignancies are usually primary tumors of breast or lung cancer, whereas osteosclerotic malignancies are usually highly associated with prostate cancer. However, this division is simple and rough, and when bone metastases from malignant tumors occur, skeletal lesions within the bone microenvironment are often complex (80). When bone metastases occur in most solid tumors, there is both an accelerated process of osteolytic destruction and bone formation and reconstruction, and bone metastases in different parts of a single patient’s body may be different, i.e. osteolytic bone destruction may occur in one part of the bone and another part of In other words, osteolytic bone destruction may occur in one part of the skeleton, while another part of the skeleton, on the contrary, may develop sclerotic osteogenic lesions or mixed bone metastases. The complexity of bone metastases along with the resistance of neoplastic cells to metastases poses a great challenge for their treatment (81).

The current standard of care for bone metastases from malignant tumors includes bisphosphonates and the RANKL inhibitor, denosumab (82), both drug types which target osteoclast inhibition (83). Bisphosphonates have been used clinically for many years, and a large amount of preclinical evidence fully demonstrates their anti-tumor cell metastatic ability (84, 85). Their action on osteoclasts leads to reduced bone resorption, which may establish a bone microenvironment unfavorable to tumor cell attachment (86). In addition, nitrogen-containing bisphosphonates can inhibit tumor angiogenesis and modulate immunity to exert indirect antitumor activity (87). Due to its good pharmacological activity and cost-effectiveness, zoledronic acid has been the standard of care for the prevention of bone metastases from malignant tumors and other SREs for nearly a decade (88), significantly improving the quality of life and survival of patients with bone metastases (89). However, the pharmacological properties of zoledronic acid have led to adverse effects (mainly acute reactions and renal impairment), making it unavailable to some patients with bone metastases (90), and until the advent of denosumab, these patients had no choice.

Denosumab was approved by the FDA in 2010 for the treatment of bone metastases from solid tumors, and has since become a breakthrough treatment for bone metastases from malignant tumors. Several clinical trials have confirmed that it is as effective as zoledronic acid (91, 92). Several large multicenter prospective double-blind randomized controlled clinical trials have shown that denosumab is effective in delaying the time to first bone metastasis, reducing the incidence of SREs, reducing pain levels in patients with bone metastases, and improving the quality of life in patients with malignancies (93, 94). However, with further clinical studies and the gradual expansion of the included population, the effectiveness of denosumab was gradually questioned (95), and several clinical studies showed that denosumab did not differ from zoledronic acid in terms of overall survival and disease progression (96). In addition, the treatment of bone metastases from malignant tumors is a long-term process, and the cost of the drug is an issue that must be considered. With conflicting clinical data from different institutional centers, more clinical studies and longer follow-up periods are needed to obtain credible evidence about the role of denosumab in malignant bone metastases, its advantages over zoledronic acid, and to analyze the various reported adverse effects from denosumab. The answers are thus both necessary and urgent.

Although clinical studies and conclusions about denosumab are still to be optimized, there is no doubt about its advantages over zoledronic acid. The emergence of denosumab brings hope to patients with bone metastases from malignant tumors combined with renal abnormalities as it is not metabolized and excreted by the kidneys (97). This makes it available for patients with bone metastases from malignant tumors treated with renal replacement therapy. This is particularly important for elderly prostate cancer patients, who are at a high risk of bone metastases. They often suffer from renal insufficiency due to malignant tumor proliferation-induced urinary tract obstruction, for which bisphosphonates are absolutely contraindicated, and who desperately need a drug that can control bone metastases (93). In addition, denosumab reduces the rate of bone resorption by competitively binding to RANKL, resulting in a durable reduction in serum calcium levels. This provides a new approach for the treatment of previously intractable hypercalcemia with bone metastases (98). Denosumab has also been reported to delay pain progression and reduce overall pain levels and analgesic drug use (99), but these reports suffer from inadequate sample sizes and are highly susceptible to subjective evaluations, which are currently unreliable.

Another question that needs to be answered is how does denosumab function in different types of tumors? Although it was approved by the FDA in 2010 for the treatment of bone metastases from solid tumors, it is not yet known whether denosumab is effective for all types of bone metastases from solid tumors (100). Clinical trials in NSCLC have shown that bone metastasis is very common in non-small cell lung cancer; one clinical trial confirmed that 50%-60% of NSCLC tumor tissues express RANKL and RANK, and the trial showed that denosumab can directly block RANKL to inhibit bone metastasis in NSCLC. However, subsequent clinical trials have indicated the opposite conclusions, since adding denosumab to standard first-line platinum-based dual therapy did not improve overall survival in advanced NSCLC. Data from different studies are conflicting, so there are questions about whether denosumab is effective in NSCLC. A more fundamental issue is that the mechanism of denosumab, which acts on osteoclasts to exert anti-metastatic effects by reducing bone resorption, may be effective in osteolytic bone metastases where osteoclast-mediated bone resorption predominates. However, there is a lack of evidence to demonstrate whether it is effective in sclerotic bone metastases where osteoblast-mediated bone formation predominates.

To minimize the impact of malignant tumor bone metastases on patients and reduce the occurrence of SREs events, the control of bone metastases often requires comprehensive treatment rather than a single therapeutic measure, and the treatment usually includes multiple treatment measures such as radiotherapy, chemotherapy, surgical treatment, immunotherapy, and palliative care. Combination therapy with denosumab is therefore a major clinical issue, most notably denosumab in combination with immunotherapy (101). RANKL, as a bridge between the skeletal and immune systems, will inevitably crosstalk with the immune system; therefore, the outcomes arising from the combination of immunotherapy and denosumab must be seriously considered. Several studies are currently underway to examine the effects of denosumab monotherapy and combination immunotherapy to assess whether the combination has a beneficial effect on progression-free survival and overall survival of patients (102).

In summary, the bone metastasis of malignant tumors has become a major challenge in tumor treatment. The various mechanisms mediating the growth of metastasis and the special skeletal microenvironment bring great challenges to the treatment of bone metastasis, and the emergence of denosumab provides a new path for the treatment of bone metastasis. However, since the understanding of denosumab is not yet perfect, more research is needed in the future to explore the great therapeutic potential of denosumab and provide a more solid and rational basis for clinical use.

## Author contributions

BS, JL, CM, and YZ conceived the idea for the study and provided critical revision of the manuscript. LS, DH, and CM collected the information. BS, JL, and DH participated in study design, supervising, writing and drafting of the manuscript. All authors contributed to the article and approved the submitted version.
